# Supplementary material for: Inclusion of cGAMP within virus‐like particle vaccines enhances their immunogenicity
Source: EMBO Rep. 2021 Jun 18;22(8):e52447. doi: 10.15252/embr.202152447 (PMC8339669; doi:10.15252/embr.202152447)
Supplement: Supplementary file 1 — Expanded View Figures PDF [file EMBR-22-e52447-s002.pdf]

Expanded View Figures

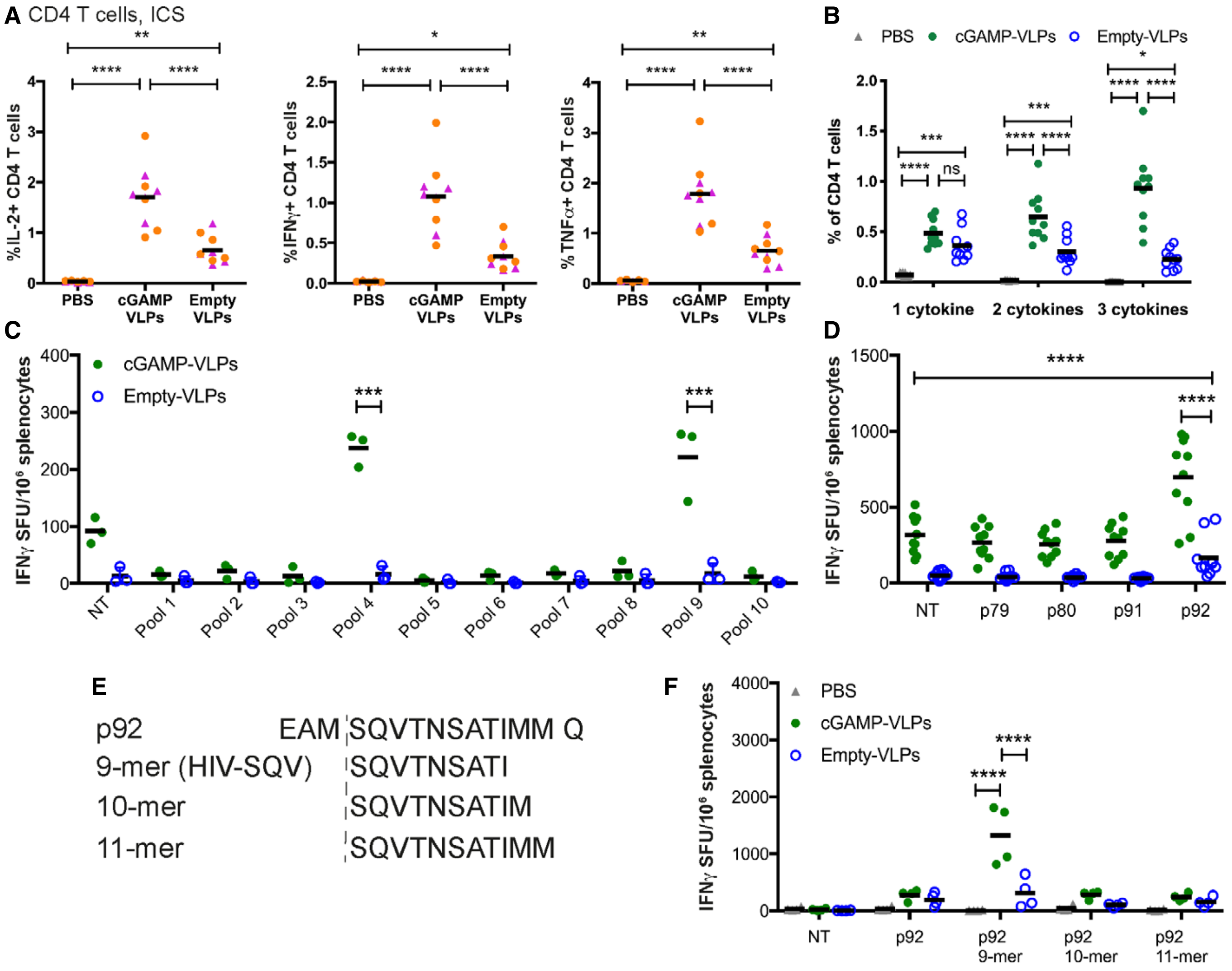

Figure EV1. cGAMP-VLPs enhance T-cell responses.

C57BL/6 mice were injected with cGAMP-VLPs, Empty-VLPs or PBS as a control *via* the intra-muscular route. 14 days later, antigen-specific T-cell responses were assessed.

- A, B BMMCs from C57BL/6 mice were pulsed overnight with Empty-VLPs and used to stimulate cells from spleens of immunised mice. Cells were co-cultured for 6 h prior to evaluation of CD4 T-cell responses by ICS. CD4 T cells were gated as shown in Fig 2A. The percentage of total CD4 T cells producing each cytokine is shown in (A), and the percentage of CD4 T cells co-producing 1, 2 or 3 cytokines is shown in (B).
- C Using a panel of 100 15-mer peptides spanning the HIV-1 Gag protein, we designed ten pools of 25 peptides so that each peptide was present in two pools and with minimal overlap between the pools. Cells from the spleens of immunised mice were stimulated for 24 h with these peptide pools and responses were read out by IFNγ ELISPOT assay. NT: not treated
- D The peptides that were common between pools 4 and 9 (p79, p80, p91, p92) were tested individually.
- E Using NetMHC, we identified a 9-mer, a 10-mer and an 11-mer in p92 as predicted strong binders to H2-D^b.
- F Splenocytes from immunised mice were stimulated with the four versions of p92 shown in (E).

Data information: Data in (A) and (B) are pooled from two independent experiments. A total of 10 mice was analysed per condition. Data in (C) and (F) are from a single experiment using three (C) or four (F) animals per group. Pooled data from two independent experiments including a total of 10 mice per group are shown in (D). In (A–D) and (F), each symbol corresponds to one animal (colour-coded by experiment in (A)) and the mean is shown. Statistical analyses were done using a 2-way ANOVA followed by Tukey's multiple comparisons test, showing only selected comparisons. *$P < 0.05$; **$P < 0.01$; ***$P < 0.001$; ****$P < 0.0001$.

© 2021 The Authors

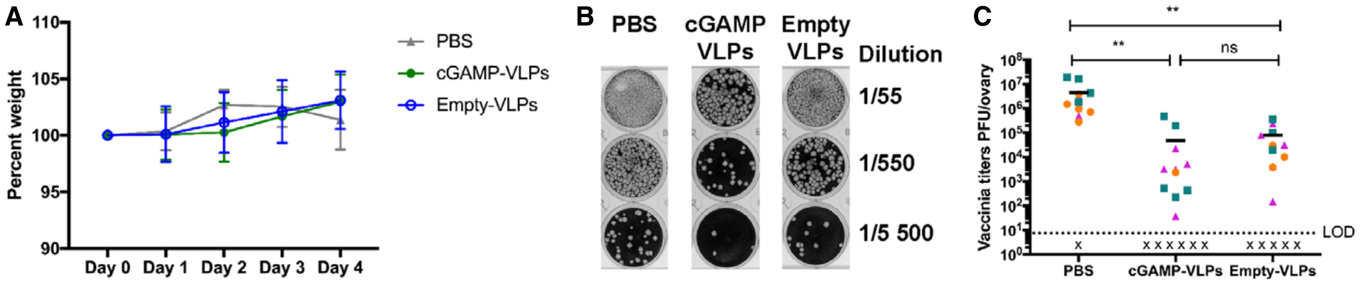

Figure EV2. Evaluation of cGAMP-VLP-elicited protection in a vaccinia virus challenge model.

Female C57BL/6 mice were injected with cGAMP-VLPs, Empty-VLPs or PBS as a control *via* the intra-muscular route. One month later, mice were infected with 10^6 PFU of a vaccinia virus expressing HIV-Gag (vVK1) by intra-peritoneal inoculation.

A Weight loss was monitored over the course of infection and is shown as a percentage of weight prior to infection.

B, C Five days after infection, virus titres in the ovaries were quantified by plaque assay. A representative example of the plaque assay is shown in (B), and pooled data from three independent experiments including a total of 12–17 mice per group are shown in (C).

Data information: A total of 12 mice (PBS) and 17 mice/group (cGAMP-VLPs and Empty-VLPs) were used in three independent experiments. In (A), mean and SD of pooled data are shown. In (C), each symbol represents data from an individual animal and colours indicate different experiments. Horizontal lines show the mean. X = sample below limit of detection (LOD). Statistical analyses were done using a 2-way ANOVA followed by Tukey's multiple comparisons test. ns $P \geq 0.05$; $^{**}P < 0.01$.

© 2021 The Authors

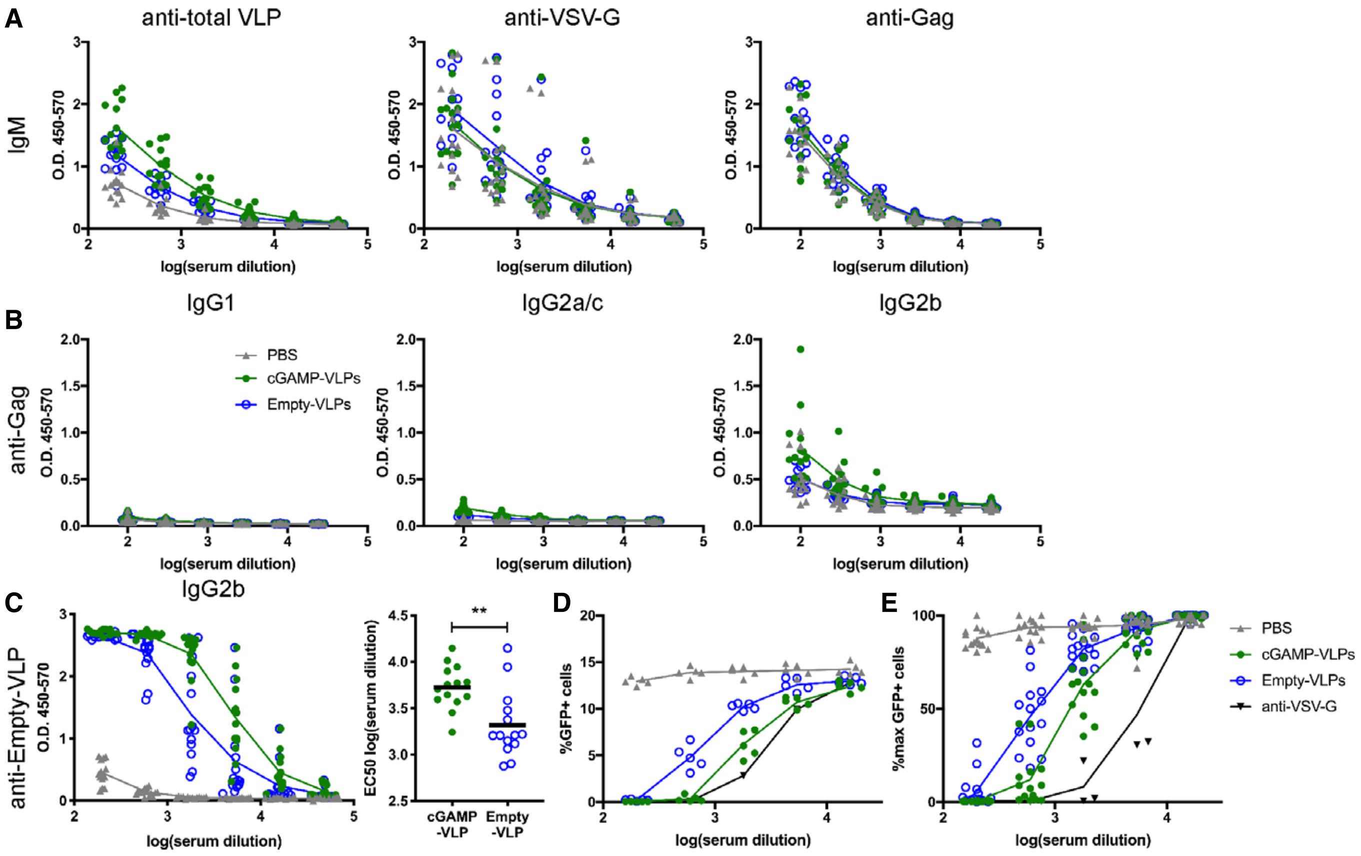

Figure EV3. Immunisation with VLPs containing cGAMP increases neutralising antibody responses.

C57BL/6 mice were injected with cGAMP-VLPs, Empty-VLPs or PBS as a control *via* the intra-muscular route. 14 days later, serum antibody responses were evaluated.

A IgM responses. ELISA plates were coated with lysate from cGAMP-VLPs, recombinant VSV-G protein or recombinant HIV-1 Gag protein. IgM antibodies specific for these proteins were measured in sera from immunised mice. The optical density at increasing serum dilutions is shown.

B Gag-specific antibody responses. ELISA plates were coated with recombinant HIV-1 Gag protein. Antibodies of different isotypes specific for this protein were measured in sera from immunised mice by ELISA. The optical density at increasing serum dilutions is shown.

C cGAMP-VLPs enhance production of anti-Empty-VLP antibodies. ELISA plates were coated with lysates from Empty-VLPs. IgG2b antibodies specific for these lysates were measured in sera from immunised mice. The optical density at increasing serum dilutions is shown on the left, and the EC50 is on the right.

D, E cGAMP-VLPs enhance production of anti-VSV-G neutralising antibodies. Serial dilutions of individual sera were incubated with VSV-G pseudotyped HIV-1-GFP for 90 min at 37°C before infection of HEK293 cells. As a control, dilutions of the anti-VSV-G neutralising antibody 8G5F11 were tested in parallel. After 2 days, infection was measured by quantifying GFP^+ cells by flow cytometry. Data from a representative experiment are shown in (D). In (E), pooled data from three independent experiments including a total of 14 mice per condition are shown. For each experiment, the infection rate was normalised by setting the highest observed proportion of GFP^+ cells to 100%.

Data information: In (A–C) and (E), data are pooled from three independent experiments. A total of 14 mice was analysed per condition. Symbols show data from individual animals, and horizontal lines show the mean. Statistical analyses were done using a Kruskal–Wallis test followed by Dunn's multiple comparisons test (C). $^{**}P < 0.01$.

 © 2021 The Authors

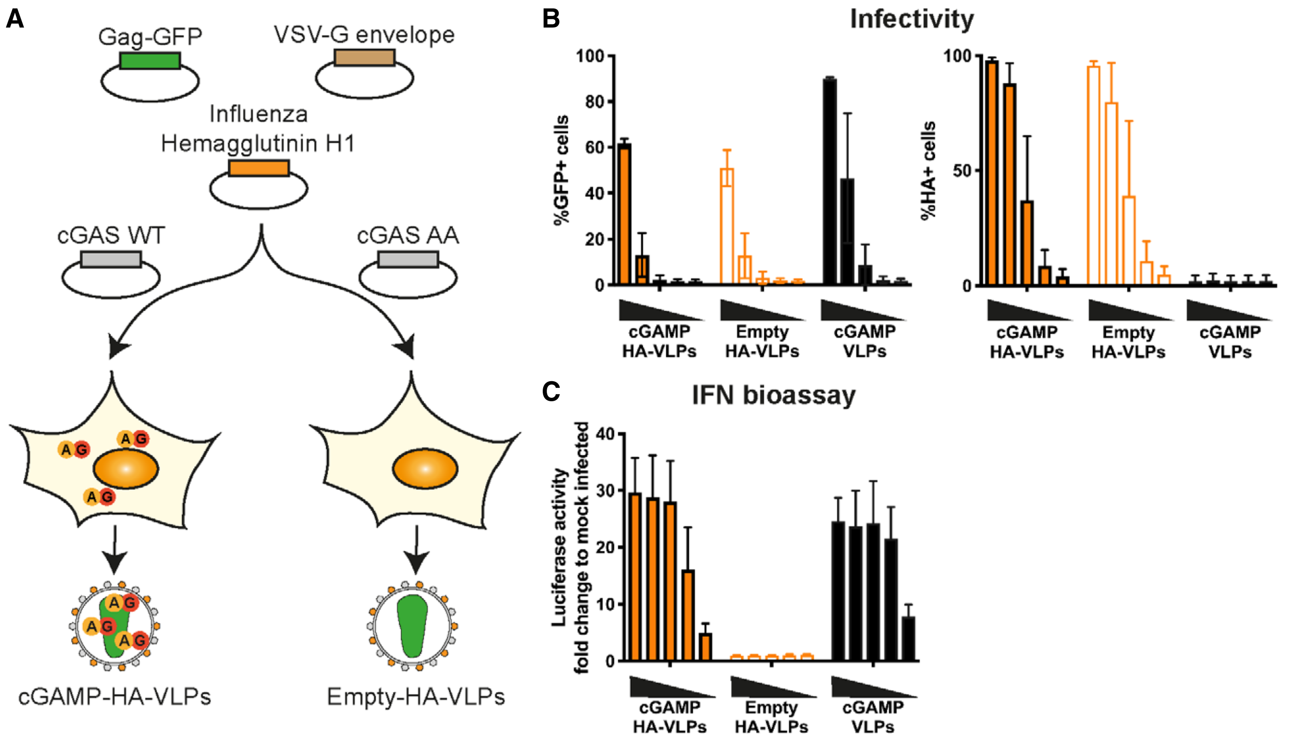

Figure EV4. Pseudotyping of cGAMP-VLPs with IAV Haemagglutinin (HA).

A Schematic representation of cGAMP-HA-VLP and Empty-HA-VLP production. HEK293T cells were transfected with plasmids encoding HIV-1 Gag-GFP, VSV-G envelope and IAV HA. cGAMP-HA-VLPs were collected from cells co-expressing cGAS WT and Empty-HA-VLPs from cells co-expressing catalytically inactive cGAS AA.

B IAV HA is present in HA-VLPs. HEK293 cells were infected with decreasing amounts of cGAMP-HA-VLPs and Empty-HA-VLPs (1/5 serial dilutions starting at 2 μl of VLP stocks per well). Infection was monitored 24 h later by quantifying GFP^+ and HA^+ cells by flow cytometry. cGAMP-VLPs were used for comparison.

C cGAMP-HA-VLPs induce a similar IFN-I response in infected cells compared with cGAMP-VLPs. Supernatants from infected cells shown in (B) were tested for the presence of IFN-I as shown in Fig 1C.

Data information: Data in (B) and (C) are pooled from three independent HA-VLP productions tested in infectivity and IFN-I bioassays; mean and SD are shown.

Figure EV5. SARS-CoV-2 Microneutralisation assay.

A, B Using serum samples from Fig 7B, antibody titres capable of neutralising SARS-CoV-2 were determined by microneutralisation (MN) assay. No serum treatment and the neutralising antibody EY6A served as negative and positive controls, respectively, and technical triplicates were performed. Panel (A) shows the raw data using a 1:10 dilution of serum samples. (B) shows the percentage of infected foci relative to the negative control for serial serum or EY6A dilutions. Each line represents one animal, and data points are mean with SD of three technical replicates. Dotted lines show 100 and 50% infection. Data are from one of two experiments each including six animals per group.

© 2021 The Authors

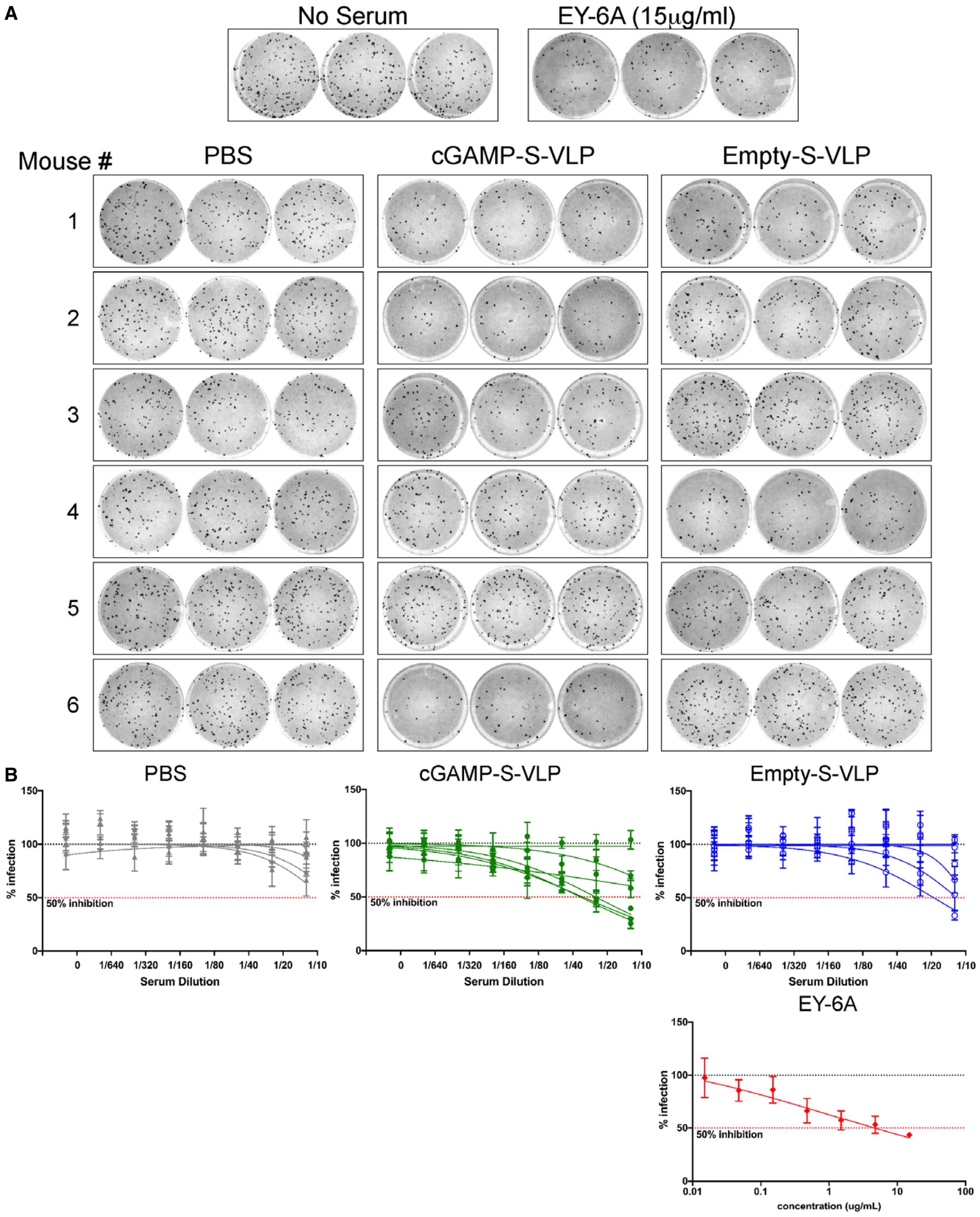

Figure EV5.

 © 2021 The Authors
